# Representation of Women Among Editors in Chief of Leading Medical Journals

**DOI:** 10.1001/jamanetworkopen.2021.23026

**Published:** 2021-09-08

**Authors:** Ana-Catarina Pinho-Gomes, Amy Vassallo, Kelly Thompson, Kate Womersley, Robyn Norton, Mark Woodward

**Affiliations:** 1 The George Institute for Global Health, Imperial College London, London, United Kingdom; 2 School of Population Health and Environmental Sciences, Faculty of Life Sciences abd Medicine, King’s College London, London, United Kingdom; 3 The George Institute for Global Health, University of New South Wales, Sydney, New South Wales, Australia

## Abstract

**Importance:**

Women remain underrepresented among editors of scientific journals, particularly in senior positions. However, to what extent this applies to medical journals of different specialties remains unclear.

**Objective:**

To investigate the gender distribution of the editors in chief at leading medical journals.

**Design, Setting, and Participants:**

Cross-sectional study of the editors in chief at the top 10 international medical journals of 41 categories related to the medical specialties of the Clarivate Analytics Web of Science Journal Citation Reports in 2019.

**Main Outcomes and Measures:**

Proportion of women as editors in chief.

**Results:**

This study found that, overall, women represented 21% (94 of 44) of the editors in chief, with wide variation across medical specialties from 0% to 82%. There were 5 categories for which none of the editors in chief were women (dentistry, oral surgery and medicine; allergy; psychiatry; anesthesiology; and ophthalmology) and only 3 categories for which women outnumbered men as editors in chief (primary health care, microbiology, and genetics and heredity). In 27 of the 41 categories, women represented less than a third of the editors in chief (eg, 1 of 10 for critical care medicine, 2 of 10 for gastroenterology and hepatology, and 3 of 10 for endocrinology and metabolism).

**Conclusions and Relevance:**

This study found that women are underrepresented among editors in chief of leading medical journals. For the benefit of medical research, a joint effort from editorial boards, publishers, authors, and academic institutions is required to address this gender gap.

## Key Points

**Question:**

Are women and men equally represented among editors in chief of leading medical journals?

**Findings:**

This cross-sectional study found that, overall, women represent only about 1 in 5 editors in chief at top-ranked medical journals, with wide variation from 0 to 82% across medical specialties.

**Meaning:**

A serious commitment from authors, editors, publishers, and the medical scientific community is required to tackle longstanding structural barriers and biases that underpin women’s underrepresentation in senior leadership roles in medical journals.

## Teaser

This cross-sectional study investigates the gender distribution of the editors in chief at leading medical journals.

## Introduction

Despite the gradual increase in the representation of women as physicians in many medical specialties over the past 50 years,^1,2^ women remain underrepresented as authors in medical journals.^3^ There is compelling evidence of a gender gap in research, in general (for instance, gender imbalances in positions of leadership and influence, success rates for major grants and fellowships, and authorship of articles).^4,5,6^ However, raising awareness has not been associated with substantial improvements. In fact, the gender gap in senior authorship positions, which are often held by the most senior author and are also the most impactful on career progression, appears to be widening.^7,8^ The COVID-19 pandemic seems to have worsened the preexisting gender bias in the authorship of journal articles, particularly those related to COVID-19,^9,10^ with far-reaching consequences across multiple medical specialties and in preprints.^11,12^ The underlying reasons are likely multifactorial and may include societal values that still preferentially attribute informal care responsibilities to women,^13^ the fact that COVID-19–related research may be shaped and led by senior academics who remain predominantly men,^14^ or the fact that the pandemic may have exacerbated women’s already greater teaching commitments in view of the need to transition to remote teaching.^15^

Journals have a key role to play in promoting gender balance and equality in authorship by adopting policies that promote women’s representation (eg, gender quotas) or that actively address barriers faced by women as authors (eg, flexible working patterns or ensuring adequate administrative support). However, gender inequalities in editorial boards may compromise engagement with such policies. Although editorial boards vary substantially in size and remit, all journals, irrespective of specialization, have at least 1 editor in chief, to oversee the production of content for publications. Editors in chief are expected to be experienced editors with good decision-making and leadership skills,^16,17^ characteristics that are perceived to be more associated with men than women.^18^ Therefore, this study investigated whether gender imbalances were present in the role of editors in chief of the top 10 journals of 41 categories related to the medical specialties of the Clarivate Analytics Web of Science Journal Citation Reports in 2019.

## Methods

This cross-sectional study investigated the gender distribution of the editors in chief of high-impact medical journals. It was conducted in line with the Strengthening the Reporting of Observational Studies in Epidemiology (STROBE) reporting guideline.^19^ Ethical approval and informed consent were waived by the institutional review board of Imperial College London because this study was based on publicly available data.

Journal categories related to medical specialties were identified in the Clarivate Analytics Web of Science Journal Citation Reports 2019. Categories related to nonphysician health professions (eg, nursing or allied health) and basic sciences related to medicine (eg, physiology or biology) were excluded. Categories referring to medical specialties associated with basic sciences (eg, medical microbiology or clinical genetics) were included in the study. eTable 1 in the Supplement lists the excluded categories. Journals within each category were ranked according to the most recent journal impact factor. The top 10 journals in each of the categories were included in the study. The outcome of this study was the proportion of women as editors in chief in each category of medical journals.

### Statistical Analysis

The gender of the editor(s) in chief of each journal was determined based on information available on the journal website, as of April 2021. Gender identity was determined according to pronouns used to describe the editor in their biography, name, and photography. When this information was not available on the journal website, the personal page of the editor in chief on the website of the affiliation(s) was consulted. Gender was assigned using the binary terms: woman or man. Data were abstracted manually by 1 researcher (A.C.P.G.). When assignment of gender was not clear using publicly available data, the editorial offices were contacted for clarification. When the role of editor in chief was shared by more than 1 person, all editors in chief were included in the analysis. All analyses were performed using Microsoft Excel.

The percentage of women as editors in chief was computed for each category of medical journals as the number of women editors in chief divided by the total number of editors in chief for that category.

## Results

A total of 41 journal categories were included, with a total of 410 journals and 444 editors in chief (eTable 2 in the Supplement). Overall, there were 29 journals with more than 1 editor in chief. All editors in chief were assigned as either a woman or man, no they or them pronouns were used in editor-in-chief biographies. The mean proportion of women as editor in chief was 21% (94 of 444), with a wide variation across categories from 0% to 82% (9 of 11) (Figure). There were 5 categories in which none of the editors in chief were women (dentistry, oral surgery, and medicine; allergy; psychiatry; anesthesiology; and ophthalmology). From our selected categories, there were only 3 categories in which women outnumbered men as editors in chief (primary health care [7 of 10], microbiology [10 of 15], and genetics and heredity [9 of 11]), and 1 category in which women and men were evenly distributed (medicine, internal and general [5 of 10]). In 27 of the 41 categories, women represented less than a third of the editors in chief (eg, 1 of 10 for critical care medicine, 2 of 10 for gastroenterology and hepatology, and 3 of 10 for endocrinology and metabolism).

**Figure.**
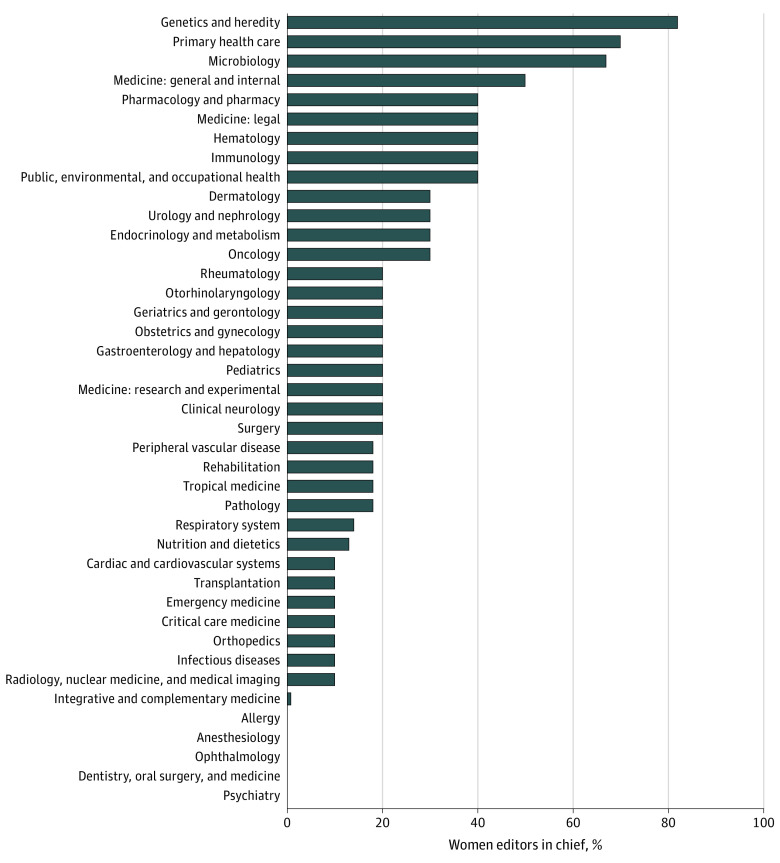
Percentage of Women as Editors in Chief in the Top 10 Highest–Impact Factor Journals in Each Category of Medical Journals

## Discussion

This study found that women are significantly underrepresented as editors in chief in comparison with men because they account for only 1 in 5 editors in chief of leading medical journals. To the best of our knowledge, this study included, thus far, the largest number of journals and editors in chief from a wide range of high-impact medical journals. The underrepresentation of women as editors in chief is broadly comparable to previous studies, thus indicating that little progress has been made. A study published 10 years ago found that only 10 of 63 editors in chief (16%) of medical journals were women, with not a single woman as editor in chief in 5 categories (critical care; anesthesiology; orthopedics; ophthalmology; and radiology, nuclear medicine, and medical imaging).^20^ Even in women’s health journals, in which women’s representation might be expected to match or even exceed men’s representation, women occupied only 54 of 132 editor-in-chief positions (41%) in 2019.^21^ However, these stark gender inequalities are not restricted to medical journals. Several studies have documented the underrepresentation of women on editorial boards in diverse fields, such as ecology and evolution,^22^ mathematics,^23^ and science in general.^24^

This poor representation of women as editors in chief and on editorial boards, in general, does not match the gradual increase in women’s representation in medicine over the past 6 decades.^1^ In the UK, women made up 48% of all licensed physicians in 2020, which reflects an increase of more than a quarter (27%) since 2012. In 2019, more than one-third (36%) of the active physicians in the United States were women, with men outnumbering women in 37 of 47 specialties (79%).^25^ The percentages of women ranged from a high of 64% in pediatrics to a low of 6% in orthopedic surgery. However, a similar increase in the representation of women in academic medicine is yet to be seen.^26^ On the other hand, the representation of women in some journal categories was equal or higher than 50% (eg, microbiology, primary health care, genetics and heredity), thus demonstrating that gender equality is achievable. Furthermore, this may not necessarily reflect women’s representation among physicians in those specialties. For instance, women represented 70% of editors in chief in primary care but they account for 41% of general practitioners in the United States.^25^ Other medical specialties should follow the example of primary care journals in promoting gender equality among their editors in chief.

The underlying reasons for the persistently low representation of women in academia, in general, and as editors in chief, in particular, are likely multifactorial. First, there is evidence that women are less likely to accept invitations to serve on journal editorial boards than men, which may be related to the fact that editorial positions are often expected to overlap with clinical or academic commitments.^22^ Indeed, an unequal distribution of teaching, pastoral care, and administrative responsibilities between women and men may limit women’s availability for research and other leadership roles.^15^ In the UK, on average, women carry out 60% more unpaid work than men. Women spend around twice as much time on unpaid cooking, childcare, and housework than men, with transport (driving self and others) being the only area in which men do more unpaid work than women.^27^ This unbalanced share of unpaid work may restrict women’s ability to take up leadership roles, such as editor in chief, and, ultimately, jeopardize their career progression.^28^ Second, women are more likely than men to take career breaks, such as maternity leave, which may further hinder career progression.^29^ These work-life interferences, together with gendered family views, may explain why women are more than men likely to experience precarious work conditions at early stages of their academic careers.^30,31^ Third, unconscious gender bias may underpin the undervaluing of women’s academic achievements^32^ and may fuel the preconception that women are unfit for senior leadership roles, including editor in chief.^33^ Fourth, the underrepresentation of women is self-perpetuating because women lack adequate mentors and role models to help them climb the career ladder.^34^ Fifth, denial or lack of awareness of gender bias by men, who still occupy the majority of senior positions in academic institutions, may be a significant deterrent to implementing policies to tackle systemic barriers that perpetuate gender inequalities.^35^

Increasing women’s representation at the top of editorial boards is critical to address longstanding inequalities across the entire scholarly publication process and in academia, in general. Indeed, having a woman editor in chief has been found to be positively associated with an increased presence of women in editorial boards and advisory boards,^24,36^ as well as in peer review.^37^ Because editors in chief are often selected from editorial boards or have experience as associate or section editors, addressing women’s underrepresentation in editorial boards seems a priority to foster gender parity at the level of editor in chief.^38^ Elsevier has been a contemporary leader in promoting gender diversity and inclusion, perhaps owing to the recent appointment of their first woman CEO in Elsevier’s 140-year history. Their report “The Researcher Journey Through a Gender Lens” is an example of accountability and transparency that other publishers should follow.^39^ It demonstrates their commitment to fulfil a global responsibility to support the United Nations’ Sustainable Development Goal 5, which aims to achieve gender equality and empower all women and girls.^40^ This example illustrates the importance of overcoming structural and cultural barriers that perpetuate male dominance in editorial boards of medical journals.

### Limitations

This study has some limitations. First, it relied on information publicly available on websites, which may not be up to date, particularly if there were changes around the time of data collection. Second, data collection may not have captured nonbinary gender identities that were not included as pronouns in editor-in-chief biographies. As investigations into this issue continue, collection of self-reported gender identity data could be prioritized to produce results that are reflective of all those working in the field of medicine. Third, it is a cross-sectional analysis that does not take into account trends over time. Although this would have been of interest, there is no information consistently publicly available on past editorial boards. Fourth, it ranked journals according to their impact factor, which is an accepted, yet limited, metric to identify the leading and influential journals in each category.^41^ However, it is unlikely that those limitations had a material impact on the key findings of this study regarding the disproportionate lack of women among editors in chief of leading medical journals.

## Conclusions

This cross-sectional study found that women are underrepresented as editors in chief of leading medical journals. A serious commitment to stem the deep-rooted issue of gender bias is required from all stakeholders, including members of editorial boards, publishers, authors, and academic institutions.^42^ For instance, providing training to editors and other editorial staff on inclusion and diversity as well as on unconscious gender bias could be an effective strategy.^43^ Journal editors should advocate for policies that promote gender equality and encourage publishers to make real-time data on gender statistics for submissions and publications, reviews, and editorial functions at all levels publicly available. In addition, the wider medical community also has a key role to play in addressing barriers that hinder women’s careers in science and academia, which often start in medical school and continue throughout their postgraduate medical training and beyond.^43,44^ Publishers, editors, authors, and academic institutions need to work collaboratively to implement solutions to promote gender-inclusive research because medicine, and science in general, have much to gain from gender balance and fairness.

## Notes

Supplement. **eTable 1.** List of Excluded Categories **eTable 2.** Percentage of Women Among Editors in Chief in Each of the Categories of Medical Journals
