# Supplementary material for: Representation of Women Among Editors in Chief of Leading Medical Journals
Source: JAMA Netw Open. 2021 Sep 8;4(9):e2123026. doi: 10.1001/jamanetworkopen.2021.23026 (PMC8427369; doi:10.1001/jamanetworkopen.2021.23026)
Supplement: Supplement. — eTable 1. List of Excluded Categories eTable 2. Percentage of Women Among Editors in Chief in Each of the Categories of Medical Journals [file jamanetwopen-e2123026-s001.pdf]

Supplemental Online Content

Pinho-Gomes AC, Vassallo A, Thompson K, Womersley K, Norton R, Woodward M. Representation of women among editors in chief of leading medical journals. *JAMA Netw Open*. 2021;4(9):e2123026. doi:10.1001/jamanetworkopen.2021.23026

eTable 1. List of Excluded Categories

eTable 2. Percentage of Women Among Editors in Chief in Each of the Categories of Medical Journals

This supplemental material has been provided by the authors to give readers additional information about their work.

© 2021 Pinho-Gomes AC et al. *JAMA Network Open.*

eTable 1. List of excluded categories

MATHEMATICS
MATERIALS SCIENCE, MULTIDISCIPLINARY
BIOCHEMISTRY & MOLECULAR BIOLOGY
NEUROSCIENCES
ENGINEERING, ELECTRICAL & ELECTRONIC
ENVIRONMENTAL SCIENCES
MATHEMATICS, APPLIED
PLANT SCIENCES
GEOSCIENCES, MULTIDISCIPLINARY
CELL BIOLOGY
CHEMISTRY, MULTIDISCIPLINARY
ECOLOGY
ZOOLOGY
CHEMISTRY, PHYSICAL
BIOTECHNOLOGY & APPLIED MICROBIOLOGY
COMPUTER SCIENCE, INFORMATION SYSTEMS
PHYSICS, APPLIED
ENGINEERING, CHEMICAL
VETERINARY SCIENCES
FOOD SCIENCE & TECHNOLOGY
COMPUTER SCIENCE, ARTIFICIAL INTELLIGENCE
MECHANICS
ENGINEERING, CIVIL
ENGINEERING, MECHANICAL
STATISTICS & PROBABILITY
NURSING
ENERGY & FUELS
COMPUTER SCIENCE, INTERDISCIPLINARY APPLICATIONS
COMPUTER SCIENCE, SOFTWARE ENGINEERING
COMPUTER SCIENCE, THEORY & METHODS

© 2021 Pinho-Gomes AC et al. *JAMA Network Open.*

MARINE & FRESHWATER BIOLOGY
MATHEMATICS, INTERDISCIPLINARY APPLICATIONS
NANOSCIENCE & NANOTECHNOLOGY
HEALTH CARE SCIENCES & SERVICES
ENTOMOLOGY
OPTICS
WATER RESOURCES
BIOLOGY
METEOROLOGY & ATMOSPHERIC SCIENCES
TOXICOLOGY
AGRONOMY
ENGINEERING, MULTIDISCIPLINARY
TELECOMMUNICATIONS
POLYMER SCIENCE
ENGINEERING, BIOMEDICAL
CHEMISTRY, ANALYTICAL
GEOCHEMISTRY & GEOPHYSICS
PHYSICS, MULTIDISCIPLINARY
SPORT SCIENCES
OPERATIONS RESEARCH & MANAGEMENT SCIENCE
PHYSIOLOGY
METALLURGY & METALLURGICAL ENGINEERING
PSYCHOLOGY
BIOCHEMICAL RESEARCH METHODS
BIOPHYSICS
CHEMISTRY, APPLIED
MULTIDISCIPLINARY SCIENCES
PHYSICS, CONDENSED MATTER
ASTRONOMY & ASTROPHYSICS
FORESTRY
OCEANOGRAPHY
INSTRUMENTS & INSTRUMENTATION
AGRICULTURE, DAIRY & ANIMAL SCIENCE

© 2021 Pinho-Gomes AC et al. *JAMA Network Open.*

AUTOMATION & CONTROL SYSTEMS
CONSTRUCTION & BUILDING TECHNOLOGY
HISTORY & PHILOSOPHY OF SCIENCE
CHEMISTRY, MEDICINAL
THERMODYNAMICS
MATHEMATICAL & COMPUTATIONAL BIOLOGY
AGRICULTURE, MULTIDISCIPLINARY
BIODIVERSITY CONSERVATION
CHEMISTRY, ORGANIC
PALEONTOLOGY
PHYSICS, MATHEMATICAL
BEHAVIORAL SCIENCES
COMPUTER SCIENCE, HARDWARE & ARCHITECTURE
ENGINEERING, ENVIRONMENTAL
FISHERIES
EVOLUTIONARY BIOLOGY
ENGINEERING, MANUFACTURING
GEOGRAPHY, PHYSICAL
ENGINEERING, INDUSTRIAL
GEOLOGY
CHEMISTRY, INORGANIC & NUCLEAR
EDUCATION, SCIENTIFIC DISCIPLINES
SPECTROSCOPY
DEVELOPMENTAL BIOLOGY
GREEN & SUSTAINABLE SCIENCE & TECHNOLOGY
ENGINEERING, GEOLOGICAL
MATERIALS SCIENCE, BIOMATERIALS
PARASITOLOGY
SOIL SCIENCE
PHYSICS, ATOMIC, MOLECULAR & CHEMICAL
VIROLOGY
HORTICULTURE
TRANSPORTATION SCIENCE & TECHNOLOGY

© 2021 Pinho-Gomes AC et al. *JAMA Network Open.*

NUCLEAR SCIENCE & TECHNOLOGY
PHYSICS, FLUIDS & PLASMAS
MATERIALS SCIENCE, CHARACTERIZATION & TESTING
ACOUSTICS
ENGINEERING, AEROSPACE
MINERALOGY
REMOTE SENSING
CELL & TISSUE ENGINEERING
MEDICAL LABORATORY TECHNOLOGY
MYCOLOGY
PHYSICS, PARTICLES & FIELDS
REPRODUCTIVE BIOLOGY
INTEGRATIVE & COMPLEMENTARY MEDICINE
MATERIALS SCIENCE, CERAMICS
ORNITHOLOGY
ROBOTICS
AUDIOLOGY & SPEECH-LANGUAGE PATHOLOGY
ELECTROCHEMISTRY
IMAGING SCIENCE & PHOTOGRAPHIC TECHNOLOGY
MEDICAL INFORMATICS
CRYSTALLOGRAPHY
MATERIALS SCIENCE, COMPOSITES
MATERIALS SCIENCE, TEXTILES
COMPUTER SCIENCE, CYBERNETICS
LIMNOLOGY
AGRICULTURAL ECONOMICS & POLICY
ANATOMY & MORPHOLOGY
LOGIC
MATERIALS SCIENCE, COATINGS & FILMS
MATERIALS SCIENCE, PAPER & WOOD
MINING & MINERAL PROCESSING
SUBSTANCE ABUSE
ENGINEERING, PETROLEUM

© 2021 Pinho-Gomes AC et al. *JAMA Network Open.*

PHYSICS, NUCLEAR
QUANTUM SCIENCE & TECHNOLOGY
MEDICAL ETHICS
ENGINEERING, MARINE
ENGINEERING, OCEAN
NEUROIMAGING
AGRICULTURAL ENGINEERING
MICROSCOPY
ANDROLOGY

© 2021 Pinho-Gomes AC et al. *JAMA Network Open.*

eTable 2. Percentage of women among editors in chief in each of the categories of medical journals

Category	N editors-in-chief (% women)
PSYCHIATRY	10 (0)
DENTISTRY, ORAL SURGERY & MEDICINE	11 (0)
OPHTHALMOLOGY	10 (0)
ANESTHESIOLOGY	10 (0)
ALLERGY	12 (0)
INTEGRATIVE & COMPLEMENTARY MEDICINE	12 (1)
RADIOLOGY, NUCLEAR MEDICINE & MEDICAL IMAGING	10 (10)
INFECTIOUS DISEASES	10 (10)
ORTHOPEDICS	10 (10)
CRITICAL CARE MEDICINE	10 (10)
EMERGENCY MEDICINE	10 (10)
TRANSPLANTATION	10 (10)
CARDIAC & CARDIOVASCULAR SYSTEMS	10 (10)
NUTRITION & DIETETICS	15 (13)
RESPIRATORY SYSTEM	14 (14)
PATHOLOGY	11 (18)
TROPICAL MEDICINE	11 (18)
REHABILITATION	11 (18)
PERIPHERAL VASCULAR DISEASE	11 (18)
SURGERY	10 (20)
CLINICAL NEUROLOGY	10 (20)
MEDICINE, RESEARCH & EXPERIMENTAL	10 (20)
PEDIATRICS	10 (20)
GASTROENTEROLOGY & HEPATOLOGY	10 (20)
OBSTETRICS & GYNECOLOGY	10 (20)
GERIATRICS & GERONTOLOGY	15 (20)
OTORHINOLARYNGOLOGY	10 (20)
RHEUMATOLOGY	10 (20)
ONCOLOGY	10 (30)
ENDOCRINOLOGY & METABOLISM	10 (30)

© 2021 Pinho-Gomes AC et al. *JAMA Network Open.*

**UROLOGY & NEPHROLOGY**	10 (30)
**DERMATOLOGY**	10 (30)
**PUBLIC, ENVIRONMENTAL & OCCUPATIONAL HEALTH**	10 (40)
**IMMUNOLOGY**	10 (40)
**HEMATOLOGY**	10 (40)
**MEDICINE, LEGAL**	15 (40)
**PHARMACOLOGY & PHARMACY**	10 (40)
**MEDICINE, GENERAL & INTERNAL**	10 (50)
**MICROBIOLOGY**	15 (67)
**PRIMARY HEALTH CARE**	10 (70)
**GENETICS & HEREDITY**	11 (82)

© 2021 Pinho-Gomes AC et al. *JAMA Network Open.*
